# Cancer cells undergoing epigenetic transition show short-term resistance and are transformed into cells with medium-term resistance by drug treatment

**DOI:** 10.1038/s12276-020-0464-3

**Published:** 2020-07-13

**Authors:** Shiv Poojan, Seung-Hyun Bae, Jae-Woong Min, Eun Young Lee, Yura Song, Hee Yeon Kim, Hye Won Sim, Eun-Kyung Kang, Young-Ho Kim, Hae-Ock Lee, Yourae Hong, Woong-Yang Park, Hyonchol Jang, Kyeong-Man Hong

**Affiliations:** 1 Research Institute, National Cancer Center, 323 Ilsan-ro, Ilsandong-gu, Goyang-si, Gyeonggi-do Republic of Korea; 2 Department of Cancer Biomedical Science, National Cancer Center Graduate School of Cancer Science and Policy, Goyang, 10408 Korea; 3 Samsung Genome Institute, Samsung Medical Center, Seoul, Korea; 4 Department of Molecular Cell Biology, Sungkyunkwan University School of Medicine, Suwon, Korea; 5 Department of Health Sciences and Technology, Samsung Advanced Institute for Health Sciences & Technology, Sungkyunkwan University, Seoul, Korea

**Keywords:** Tumour heterogeneity, Epigenetics

## Abstract

To elucidate the epigenetic mechanisms of drug resistance, epigenetically reprogrammed H460 cancer cells (R-H460) were established by the transient introduction of reprogramming factors. Then, the R-H460 cells were induced to differentiate by the withdrawal of stem cell media for various durations, which resulted in differentiated R-H460 cells (dR-H460). Notably, dR-H460 cells differentiated for 13 days (13dR-H460 cells) formed a significantly greater number of colonies showing drug resistance to both cisplatin and paclitaxel, whereas the dR-H460 cells differentiated for 40 days (40dR-H460 cells) lost drug resistance; this suggests that 13dR-cancer cells present short-term resistance (less than a month). Similarly, increased drug resistance to both cisplatin and paclitaxel was observed in another R-cancer cell model prepared from N87 cells. The resistant phenotype of the cisplatin-resistant (CR) colonies obtained through cisplatin treatment was maintained for 2–3 months after drug treatment, suggesting that drug treatment transforms cells with short-term resistance into cells with medium-term resistance. In single-cell analyses, heterogeneity was not found to increase in 13dR-H460 cells, suggesting that cancer cells with short-term resistance, rather than heterogeneous cells, may confer epigenetically driven drug resistance in our reprogrammed cancer model. The epigenetically driven short-term and medium-term drug resistance mechanisms could provide new cancer-fighting strategies involving the control of cancer cells during epigenetic transition.

## Cancer chemotherapy: treatment prolongs resistance caused by DNA modification

Cancer cells that are transiently resistant to drug therapies owing to changes in their gene expression patterns can become resistant for longer durations if exposed to the drug treatments. A team led by Kyeong-Man Hong and Hyonchol Jang from the National Cancer Center in Goyang, South Korea, used cellular reprogramming technologies to induce changes in the DNA markers that regulate gene expression. Working with lung and gastric cancer cell lines, the researchers found that such epigenetic alterations caused many cells to become resistant to the chemotherapy drugs cisplatin and paclitaxel. In the absence of treatment, the cells soon lost their drug resistance. In the presence of the chemotherapeutics, however, the resistance trait lasted longer, a finding that could inform best practice for how to administer cancer-fighting agents in the face of epigenetic-driven drug resistance.

## Introduction

Drug resistance is the major cause of drug treatment failure in cancer patients, and its genetic mechanisms are well understood^1–5^. In drug resistance due to genetic mechanisms, the outgrowth of rare cancer cell variants among genetically heterogeneous cancer cells occurs after the acquisition of point mutations or copy-number changes. However, rare mutant cells that grow after the acquisition of genetic changes take a long time to show clinical resistance; additionally, there are limitations to genetic mechanisms as the cause of the rapid appearance of drug resistance after the initial drug response in many cancer patients.

Recently, epigenetic mechanisms have been posited as important factors in rapid cancer drug resistance acquisition^6–8^. However, the process of the development of drug resistance via epigenetic mechanisms has not been fully elucidated. Recently, epigenetically reprogrammed cancer (R-cancer) cells have been proposed as a powerful tool for discerning the roles of the epigenome in cancer^9^. Additionally, R-cancer cells could be a promising means of elucidating the process of drug resistance by epigenetic mechanisms.

In the present study, reprogramming factors (*OCT4*, *SOX2*, *KLF4,* and *MYC*) were transiently transfected into cancer cells in the form of mRNAs to avoid inducing permanent genomic changes during the preparation of R-cancer cells, as described previously^10^. The stem cell medium was withdrawn from the R-cancer cells for various durations to induce differentiation via epigenetic modulation, and the resulting differentiated R-cancer (dR-cancer) cells were treated with cisplatin or paclitaxel to monitor changes in drug resistance because epigenetic mechanisms have been suggested to be responsible for resistance to cisplatin^11^ and paclitaxel^12^. Additionally, the single-cell analysis of the dR-cancer cells was performed to monitor changes in their heterogeneity.

## Materials and methods

### Chemicals and cells

Trypsin/EDTA, penicillin-streptomycin, culture plates, and flasks were purchased from Gibco Thermo Fisher Scientific (Grand Island, NY, USA). Fetal bovine serum (FBS), Dulbecco’s modified Eagle’s medium (DMEM), and RPMI-1640 medium were obtained from HyClone (Logan, UT, USA). Conical 50 and 15 ml tubes were acquired from SPL (Seoul, South Korea). Culture dishes with a volume of 100 mm were obtained from Falcon (Corning, NY, USA). H460 and N87 cells were acquired from the Korean Cell Bank (Seoul, Korea), and their authentication was performed at the Research Core Center of the National Cancer Center.

### Preparation of reprogrammed cancer (R-cancer) cells

Cancer cells were reprogrammed using the Stemgent mRNA Reprogramming System (Reprocell, Beltsville, MD, USA) according to the manufacturer’s instructions with some modifications, which are described in Fig. S1.

#### Culture media employed in the present study

- Complete DMEM or RPMI medium: DMEM or RPMI-1640 medium containing 10% FBS
- Reprogramming medium (R-medium): 10 ml of Pluriton^TM^ Medium was mixed with 4 µl of Pluriton^TM^ Supplement in the Stemgent mRNA Reprogramming System (Reprocell, Beltsville, MD, USA). R-medium was prepared freshly before use.
- Conditioned-R-medium: the culture supernatant from the culture of irradiated fibroblasts (Millipore, Billerica, MA, USA) in Pluriton^TM^ Medium supplemented with bFGF (final concentration of 4 ng/ml) was mixed with Pluriton^TM^ Supplement just before use.
- Stem cell medium: mTeSR^TM^1 medium (StemCell Technologies, Vancouver, Canada)

#### Gamma irradiation of human fibroblasts and preparation of conditioned medium

Human fibroblast cells (Millipore) were seeded in a 100 mm dish in complete DMEM at 37 °C under 5% CO_2_. At 90% confluence, the cells were detached, and a total of 1 × 10^7^ cells were suspended in 50 ml DMEM without serum. The suspended cells were irradiated in a gamma radiation chamber for 5 min at 60 Grays by using a Gammacell 3000 elan irradiator (MDS Nordion, Ottawa, Canada). After suspension in freezing medium (Reprocell, Beltsville, MD, USA), the irradiated human fibroblasts were stored frozen in a liquid nitrogen tank until use.

For the preparation of conditioned medium, irradiated human fibroblasts were thawed, and 5 × 10^6^ cells in 10 ml of complete DMEM were seeded in a T75 flask. After overnight incubation, the medium was changed to Pluriton^TM^ medium supplemented with bFGF. After a further 48 h of incubation, the culture supernatant was collected, and, after the substitution of fresh Pluriton^TM^ Medium supplemented with bFGF, this process was repeated for six cycles. All of the collected culture supernatants were pooled and transferred to 15 ml conical tubes and maintained at −80 °C until use.

#### Feeder layer formation and transfer of target cancer cells onto the feeder layer

For the preparation of gelatin-coated 6-well plates, 1 ml of 0.2% gelatin (Sigma-Aldrich, St. Louis, MO, USA) in phosphate-buffered saline (PBS) was employed, and the coated plate was incubated for 30 min at 37 °C. Irradiated human fibroblasts (Millipore) were thawed and seeded in gelatin-coated plates at a density of 2.5 × 10^5^ cells in complete DMEM per well in 6-well plates. The cells were incubated overnight at 37 °C under 5% CO_2_, and R-medium was added. The prepared fibroblasts were employed as the feeder layer.

To adapt the target cancer cells to the change in the medium from complete RPMI to R-medium, H460 and N87 cells were incubated in R-medium for 5 days. Then, the adapted cancer cells were detached with trypsin-EDTA and washed with PBS. The detached cancer cells were seeded on the feeder cell layer in R-medium at a density of 2.5 × 10^5^ cells per well.

#### Transfection of reprogramming factor mRNAs into target cells on the feeder layer

On the next day (day 1), after the transfer of target cancer cells to the feeder layer, the medium was exchanged for fresh R-medium. Then, the transfection of mRNAs of *OCT4*, *SOX2*, *KLF4,* and *MYC* (reprogramming factors) was performed. The mRNA transfection complex (120 μL), which was prepared according to the manufacturer’s instructions, was added to each well in a dropwise fashion, and the transfected cells were incubated for 4 h at 37 °C under 5% CO_2_. After incubation, the mRNA transfection complex was removed by aspiration, and freshly prepared R-medium (2 ml) was added. Then, the plate was incubated overnight at 37 °C under 5% CO_2_. Transfection was repeated every day until day 8.

#### Transfer of target cancer cells on the feeder layer to CTS (CELLstart^TM^ CTS^TM^)- or CTS/Matrigel-coated plates

On day 8, a CELLstart^TM^ CTS^TM^ (CTS; Gibco Thermo Fisher Scientific)-coated (for the transfection of H460) or CTS/Matrigel-coated (for the transfection of N87) 6-well plate was prepared. For the preparation of the CTS-coated plate, 1 ml of diluted CTS (1:50 in Dulbecco’s PBS, Gibco Thermo Fisher Scientific) was added per well according to the instructions, and for the preparation of the CTS/Matrigel-coated plate, 1 ml of diluted CTS and 1 ml of diluted Matrigel solution (mixture of one vial of Matrigel stock solution [Corning] and 25 ml of Pluriton^TM^ medium) were mixed. Thereafter, 1 ml of the mixture was added per well in a 6-well plate, and the plate was incubated first on a clean bench for 30 min and then overnight at 4 °C. To transfer the target cancer cells to the prepared CTS- or CTS/Matrigel-coated plates, the cancer cells on the feeder layer were detached and seeded in a CTS-coated (for H460) or CTS/Matrigel-coated (for N87) plate in conditioned-R-medium.

#### Transfection of reprogramming factors into cancer cells on CTS- or CTS/Matrigel-coated plates

After the overnight incubation of the cancer cells on CTS- or CTS/Matrigel-coated plates, the transfection of reprogramming factors was performed as described for the cancer cells on the feeder cell layer. Conditioned R-medium was replaced every other day until the end of the transfection procedure on day 23. Typically, 6–12 R-cancer colonies were observed within 3 weeks from the initiation of transfection. Starting on day 24, B18R (0.5 mg/ml) was added to the conditioned R-medium to maintain the R-cancer cells. On day 30, distinct colonies were detached by scraping with a sterile Pasteur pipette under light microscopy on a clean bench and were transferred to freshly prepared Matrigel-coated 6-well plates in stem cell medium.

#### Maintenance of R-cancer cells

To maintain the R-cancer cells in the undifferentiated state, they were cultured in stem cell medium on six-well plates-coated with Matrigel (BD Biosciences, San Jose, CA, USA). On a daily basis, the medium was replaced. When the R-cancer cells reached 80% confluence, they were treated with Stempro Acutase (Life Technologies, Carlsbad, CA, USA) to detach them from the surface, and the cells were divided at a ratio of 1:3. For long-term maintenance, the R-cancer cells were kept frozen at −70 °C. Thawed cells were cultured in conditioned-R-medium with B18R for 24 h, after which they were cultured in stem cell medium again.

### Immunofluorescent staining with TRA-1-60 or activity staining of alkaline phosphatase for the identification of R-cancer colonies

For live cell staining with TRA-1-60, the StainAlive^TM^ TRA-1-60 antibody (DyLight 488, Stemgent) was diluted 1:100 in conditioned-R-medium supplemented with B18R. The antibody in the medium was added to the reprogramming factor-transfected colonies and incubated at 37 °C under 5% CO_2_ for 30 min. After the removal of the staining medium, fresh con-Pluriton-R-B18R medium was added, and the positive colonies were identified under fluorescence microscopy. Then, TRA-1-60-positive colonies were manually isolated by scraping with a Pasteur pipette under light microscopy on a clean bench, and the isolated colonies were transferred to CTS-coated or CTS/Matrigel-coated 6-well plates. Isolated individual colonies were maintained and expanded in mTeSR1 medium. Some of the R-cancer cells from the isolated colonies were employed for cell authentication and were stored frozen in a liquid nitrogen tank after resuspension in freezing medium (Reprocell).

To reconfirm the R-cancer cells, alkaline phosphatase staining was performed with the isolated R-cancer cells in 24-well plates as described previously^13^.

### MACS sorting and culture of R-N87 cells

For the enrichment of R-N87 cells, reprogrammed cancer cells were dissociated using Stempro^TM^ acutase (Thermo Fisher Scientific), and TRA-1-60-positive cells were sorted using the anti-TRA-1–60 MicroBead kit (Miltenyi Biotech, Auburn, CA, USA) according to the manufacturer’s instructions. To reduce cell death, 10 μM ROCK inhibitor (Miltenyi Biotech) was introduced to the cell culture medium during the process.

### Stem cell medium withdrawal from R-cancer cells and colony-formation assay for drug resistance

To induce epigenetic modulation in the R-cancer cells, the cells were differentiated by the withdrawal of the stem cell medium, mTeSR1, and its replacement with complete RPMI medium.

For the colony-formation assay, the R-cancer cells were induced to differentiate for 3, 7, 13, and 40 days. On the next day post-cell counting, after the transfer of the cells in complete RPMI medium to freshly prepared Matrigel-coated 10 mm dishes, cisplatin (5 μM, Tocris) or paclitaxel (5 nM, Tocris) was added to the differentiated R-cancer (dR-cancer) cells for 3 days. After drug treatment, the cells were maintained in complete RPMI media for 30 days. The colonies were stained with 0.5% crystal violet staining solution, and images were obtained as described previously^14^.

For the stability testing of drug resistance or expression profiling, cisplatin-resistant (CR) colonies were identified and selected under microscopy on a clean bench and maintained in complete RPMI medium.

### RNA sequencing and analysis

Total RNA was extracted with TRIzol (Life Technologies) according to the manufacturer’s instructions^15^. RNA quality and quantity were assessed by using a Bioanalyzer 2100 with RNA 6000 Nano Labchips (Agilent Technologies, Dublin, Ireland). The preparation of RNA libraries using the Truseq RNA-Seq Library Prep Kit-v2 and paired-end sequencing using the HiSeq 2500 sequencing system (Illumina, San Diego, CA, USA) were performed by Macrogen (Seoul, Korea). The RNA sequencing data were deposited in the Gene Expression Omnibus GEO database under accession number GSE139887.

Differentially expressed genes (DEGs) were filtered as previously reported^14^, except that the genes showing fragments per kilobase of transcript per million mapped reads (FPKM) values below 10 were excluded from the subsequent analysis. Ingenuity pathway analysis (IPA^®^, QIAGEN, Redwood City, CA, USA) was also performed to identify key biological functions based on the curated diseases and functional ontologies in the IPA knowledge database, as previously reported^14^. Multi-Experiment Viewer 4.9.0 (mev.tm4.org) was used for the graphic representation of the values.

### Analysis of the single-cell transcriptome

For single-cell transcriptome analysis, parental H460 cells and 13dR-H460 and 40dR-H460 cells were cultured in 75 mm flasks. Single-cell suspensions in PBS were filtered through a MACS SmartStrainer (30 μm) and loaded into a Chromium system (10x Genomics, Pleasanton, CA, USA) targeting 5000 cells, following the manufacturer’s instructions. Barcoded sequencing libraries were generated using Chromium Single Cell 3′ reagent kits (v2 chemistry) and sequenced using the HiSeq 2500 platform (Illumina).

Single-cell raw reads from the 10x Genomics platform (https://www.10xgenomics.com/) were processed with Cell Ranger (version 3.0)^16^. Thereafter, all analyses were performed using the R package SEURAT version 3.0^17^ with custom parameters in subset command: H460 with nFeature_RNA > 200, nFeature < 5000, and percent.mt < 10; 13dR-H460 with nFeature_RNA > 200, nFeature RNA < 5000, and percent.mt < 10; 40dR-H460 with nFeature_RNA > 200, nFeature RNA < 4000, and percent.mt < 10. The integration of three samples was carried out using the IntegrateData command, and dimension reduction was performed using UMAP (uniform manifold approximation and projection) with 11-dimensional data in a principal component analysis, as described previously^18^.

### Statistical analysis

The statistical analyses were performed with GraphPad Prism version 5 (GraphPad Software Inc., San Diego, CA, USA). The Mann-Whitney test was used to determine the significance of differences in the median colony count values.

## Results

### Preparation of epigenetically reprogrammed H460 (R-H460) cells by the transient introduction of reprogramming factors

**Fig. 1:**
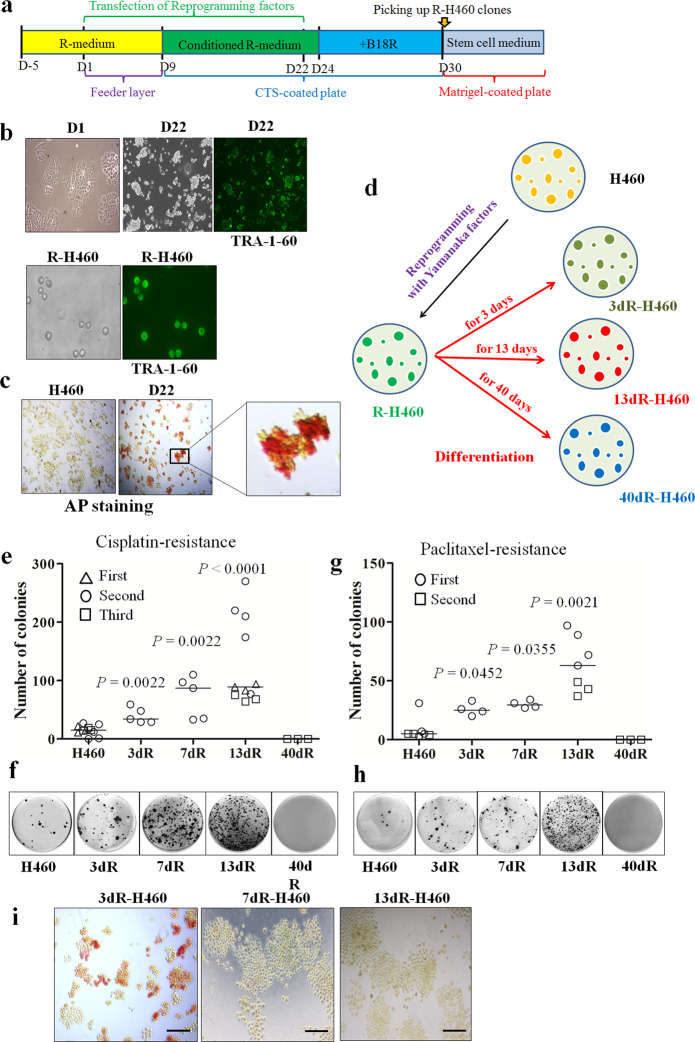
Establishment of reprogrammed H460 (R-H460) cells. **a** H460 cells were reprogrammed using the Stemgent mRNA Reprogramming System (Reprocell, Beltsville, MD, USA). Schedule for establishing R-H460 cells by transfecting the mRNAs of reprogramming factors. Reprogramming factors were transfected daily from day 1 (D1) to day 22 (D22). After the initial use of reprogramming medium (R-medium), conditioned R-medium was used from day 9 (D9) onward, and the B18R recombinant protein was added to the medium from day 24 (D24) onward. Depending on the schedule, feeder layers, CELLstart^TM^ CTS^TM^ (CTS; Gibco Thermo Fisher Scientific)-coated plates, or Matrigel-coated plates were used. The final R-cancer cell clones were picked approximately 30 days after the initiation of transfection and maintained in stem cell medium (mTeSR^TM^1; STEMCELL technologies). **b** TRA-1-60 staining for the establishment of R-H460 cells. Bright-field images of H460 cells on day 1 (D1) and day 22 (D22) after the initiation of transfection are shown, along with the TRA-1-60 status at D22 (TRA-1-60 at D22). TRA-1-60 positivity in the established R-H460 cells (R-H460) was tested again after the subculture of the R-H460 colonies (TRA-1-60 in R-H460). **c** Alkaline phosphatase staining was positive in transfected H460 cells at D22 but not in parental H460 cells. The inset shows a magnified image. **d** Scheme for the preparation of differentiated R-H460 cells (dR-H460). R-H460 cells were differentiated by the withdrawal of stem cell medium for the indicated periods of time and were referred to as dR-H460 cells (for example, if R-H460 cells were differentiated for 3 days, they were referred to as 3dR-H460 cells). **e–h** Colony-formation assay after drug treatment. dR-H460 cells were treated with cisplatin (5 μM) or paclitaxel (5 nM) for 3 days. After culture in drug-free media for approximately 30 days, the drug-resistant colonies were counted. **e**, **g** The median colony numbers and the *P*-values relative to H460 are indicated. Experiments with cisplatin and paclitaxel were repeated 3 and 2 times, respectively. **f**, **h** Representative images of the colony-formation assay. **i** Loss of alkaline phosphatase activity along with differentiation. Alkaline phosphatase was positive in some of the R-H460 cells until day 3 after the withdrawal of stem cell medium but faded out to become negative on day 13.

Reprogramming factors (*OCT4*, *SOX2*, *KLF4*, and *MYC*) in the form of mRNA were transfected into H460 cells for 22 days in reprogramming medium (R-medium) or conditioned-R-medium, and the reprogrammed cancer (R-cancer) cells were then maintained in conditioned-R-medium supplemented with B18R to obtain reprogrammed H460 cell (R-H460) clones. R-H460 clones were picked and maintained in stem cell medium (Fig. 1a). For the confirmation of R-H460 colonies during establishment, stem cell markers such as TRA-1-60 and alkaline phosphatase (AP) were tested: both TRA-1-60 and AP expression was observed in R-H460 cells but not in the parental H460 cells (Fig. 1b, c). Cell authentication of the finally established R-H460 cells confirmed the origin.

### Increased cisplatin resistance in short-term-differentiated R-H460 cells

To induce epigenetic modulation, R-H460 cells were differentiated by the changing stem cell medium to complete RPMI medium for various periods (Fig. 1a). The resulting dR-H460 cells were prepared according to different periods of differentiation induction: 3dR-H460, 7dR-H460, 13dR-H460, and 40dR-H460, corresponding to differentiation periods of 3, 7, 13, and 40 days, respectively (see the scheme shown in Fig. 1d).

For the analysis of the cisplatin resistance of the dR-H460 cells, colony-formation assays after treatment with cisplatin (5 μM, at a concentration of IC_50_) were performed on the parental H460 and dR-H460 cells. Initially, we assumed that heterogeneity would be the main reason for drug resistance in our R-cancer model, and the stem cell medium was therefore withdrawn for a long period (approximately 4 weeks) to prepare dR-H460 cells; however, we found that the cisplatin-resisatnt (CR) colony number of the dR-H460 cells was not significantly different from that of the parental H460 cells (data not shown). After the reduction of the duration of differentiation, the CR colony number of dR-H460 cells, including 3dR-H460 (*P* = 0.0022), 7dR-H460 (*P* = 0.0022), and 13dR-H460 (*P* < 0.0001), but not 40dR-H460 cells, was significantly higher than that of the parental H460 cells (Fig. 1e, f), indicating that dR-H460 cancer cells show cisplatin resistance only in short-term transitional periods (less than a month) after the differentiation induction of R-H460 cells.

### Increased paclitaxel resistance of 13dR-H460 cells and alkaline phosphatase expression in dR-H460 cells

To determine whether the dR-H460 cells showed resistance to other drugs, paclitaxel was administered to the dR-H460 cells. In colony-formation assays, significantly more paclitaxel-resistant colonies were observed in 13dR-H460 (*P* = 0.0021), 7dR-H460 (*P* = 0.0355), and 3dR-H460 cells (*P* = 0.0452) (Fig. 1g, h). Additionally, the 13dR-H460 cells showed a significantly greater number of paclitaxel-resistant colonies than the 3dR-H460 cells (*P* = 0.0061), suggesting that resistance to paclitaxel peaked at approximately 13 days after differentiation, as observed for cisplatin. The 40dR-H460 cells did not show a greater number of paclitaxel-resistant colonies than the parental H460 cells, as was the case for cisplatin.

We were concerned that the higher resistance of dR-H460 cells was due to the characteristics of the minor remnant fraction of R-H460 cells. However, alkaline phosphatase expression had already faded away in the 3dR-H460 cells, and no alkaline phosphatase expression was observed in the 7dR-H460 or 13dR-H460 cells (Fig. 1i), suggesting that the characteristics of the R-H460 cells had disappeared under differentiation induction for 7 days but only partially disappeared under induction for 3 days. If the drug-resistant colony number was highest in 3dR-H460 cells, a significant portion of which showed AP expression, the characteristics of the remnant R-H460 cells among the 3dR-H460 population could be the major reason for the increased drug resistance. In the analyses of cisplatin resistance in various dR-H460 cells, however, the 13dR-H460 cells showed a significantly greater number of drug-resistant colonies than the 3dR-H460 cells (*P* = 0.0022 for cisplatin, *P* = 0.0061 for paclitaxel), indicating that resistance to both cisplatin and paclitaxel peaked at approximately 13 days after differentiation. These results also indicated that the remnant R-H460 cells were not the main reason for the increased drug resistance of 13dR- or 3dR-H460 cells.

### Loss of drug resistance after 2–3 months of drug-free culture of CR 13dR-H460 colonies

**Fig. 2:**
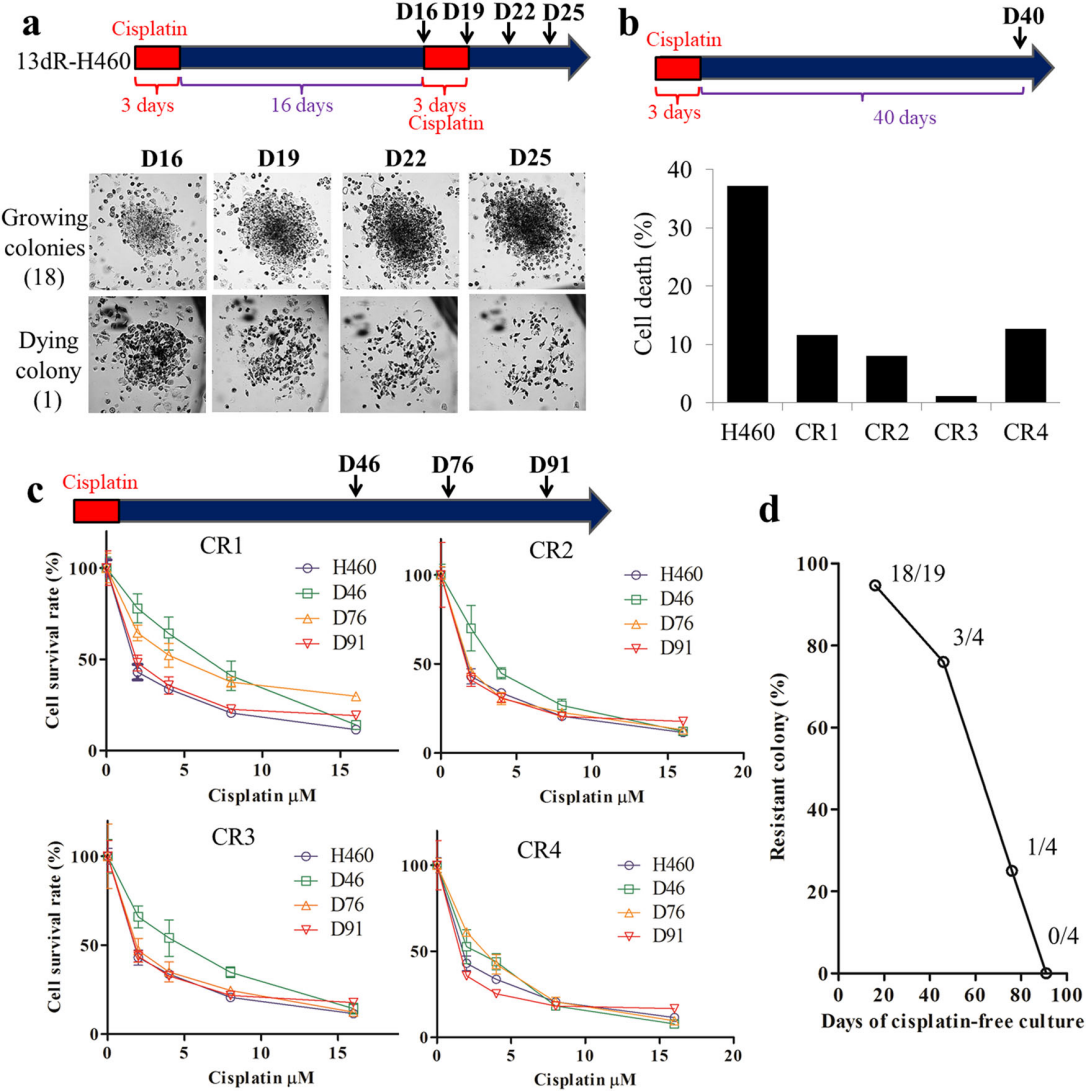
Medium-term maintenance of drug resistance in cisplatin-resistant (CR) 13dR-H460 clones. **a** Maintenance of cisplatin resistance in CR 13dR-H460 colonies under cisplatin-free culture until day 16. After the cisplatin treatment of 13dR-H460 cells, the 19 resistant colonies were marked with a pen on day 16 and were monitored by taking pictures on days 16, 19, 22, and 25 (marked as D16, D19, D22, and D25, respectively). Almost all of the resistant colonies (94.7%, 18/19) maintained cell growth or cisplatin resistance. **b** Maintenance of cisplatin resistance under cisplatin-free culture for 40 days (D40). Relative cell death was monitored by Annexin V staining with and without cisplatin treatment (5 μM) in 4 isolated CR 13dR-H460 colonies (CR1, CR2, CR3, and CR4) under cisplatin-free culture for 40 days. Only one experiment was performed. **c** Determination of cisplatin resistance (IC_50_) on days 46 (D46), 76 (D76), and 91 (D91) after selection for cisplatin resistance. The IC_50_ for cisplatin was determined in CR 13dR-H460 colonies along with the parental H460 cells. The *Y*-axis, cell survival rate (%). The *X*-axis, cisplatin concentration (μM). **d** Plot of the decrease in cisplatin resistance in CR 13dR-H460 colonies according to cisplatin-free culture days. All CR colonies from 13dR-H460 cells lost cisplatin resistance in cisplatin-free cultures over the course of 80–90 days.

After CR 13dR-H460 clones were established by cisplatin treatment for 3 days, the clones were maintained for 16 days without cisplatin. After distinct colonies formed and were identified in culture dishes, cisplatin treatment was applied again for 3 days, during which colony images were taken: among the 19 colonies, only one was not maintained by cisplatin treatment (Fig. 2a), indicating that almost all of the colonies arising after cisplatin treatment (94.7%, 18/19) retained cisplatin resistance on day 16 after cisplatin withdrawal. In a separate dish, 4 colonies were separated, which were referred to as CR1, CR2, CR3, and CR4. The CR colonies were maintained for 40 days after the first cisplatin treatment, and relative cell death was monitored with Annexin V staining with and without cisplatin treatment. All of the colonies showed lower cell death than the parental H460 cells, indicating that cisplatin resistance had been maintained for 40 days (Fig. 2b). However, cisplatin resistance was shown to be gradually lost in measuring the IC_50_ for cisplatin in each clone: one colony showed cisplatin resistance on day 76, but none showed cisplatin resistance on day 90 (Fig. 2c). Therefore, as shown in Fig. 2d, all of the initially established CR colonies from 13dR-H460 cells lost cisplatin resistance gradually over time by day 90, suggesting that cisplatin resistance in CR colonies is not a genotype but a transient phenotype. Additionally, our results suggest that the drug treatment of 13dR-H460 or short-term epigenetically transitional cells can induce medium-term (approximately 2-3 months) drug resistance.

### Preparation of reprogrammed N87 (R-N87) cells and enrichment of R-N87 cells by sorting with a TRA-1-60 antibody

**Fig. 3:**
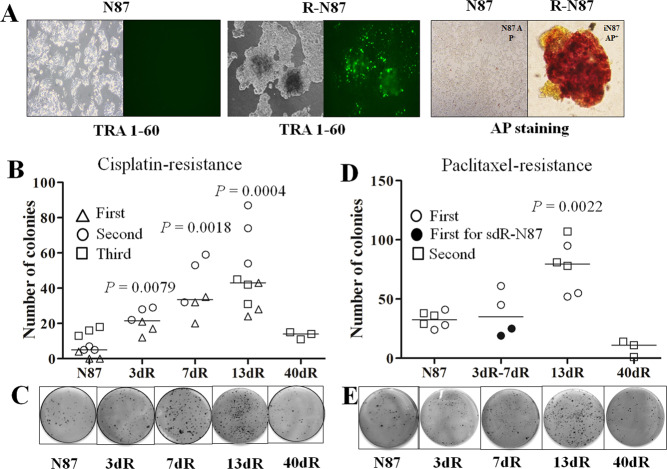
Establishment of reprogrammed N87 (R-N87) cells. **a** TRA-1-60 and alkaline phosphatase staining in R-N87 cells after the transfection of mRNAs for reprogramming factors. Positivity for both TRA-1-60 and alkaline phosphatase was observed in the established R-N87 (R-N87) cells but not in parental N87 (N87) cells. **b** Colony-formation assay after treatment with cisplatin (5 μM) in R-87 cells. The 13dR-N87 cells also showed significantly greater numbers of colonies than the 3dR-N87 cells (*P* = 0.0067). The experiment was repeated three times (first, second, and third), and the median colony numbers are indicated. **c** Representative images of colony-formation assays for cisplatin resistance in N87 and dR-N87 variants. **d** Colony-formation assay after treatment with paclitaxel (5 nM) in R-87 cells. The 13dR-N87 cells (*P* = 0.0022) showed a significantly greater number of paclitaxel-resistant colonies than did the parental N87 cells. The colony number of the 13dR-N87 cells was also significantly higher than that indicated by the combined data from 3dR-H460 (closed circle) and 7dR-H460 cells (open circle, *P* = 0.0381). The experiment was repeated two times (first and second), and the median colony numbers are indicated. (**e)** Representative images of colony-formation assays for paclitaxel resistance in N87 and dR-N87 variants.

To investigate whether drug resistance was higher in other cancer cells than in H460 cells according to the R-cancer model, we prepared R-cancer cells from N87 cells. During the establishment of R-N87 cells, TRA-1-60 and AP expression was observed in R-N87 cells, but not in the parental N87 cells (Fig. 3a). Because we could not perform all of the assays for higher drug resistance in dR-cancer cells at the same time as in the established R-N87 cells, we had to freeze the cells in aliquots and perform assays after the retrieval of the frozen R-N87 cells, as was the case for R-H460 cells. However, a significant portion (approximately 70–80%) of the retrieved R-N87 cells did not express TRA-1-60, probably due to the tendency to readily lose R-cancer characteristics after a freeze-thaw cycle, in contrast to the stable TRA-1-60-expressing R-H460 cells after a freeze-thaw cycle. Therefore, we enriched the R-N87 population by using magnetic beads coated with the TRA-1-60 antibody for sorting and employed the enriched R-N87 cells in tests for drug resistance.

### Increased cisplatin and paclitaxel resistance in 13dR-N87 cells

After stem cell medium withdrawal from the TRA-1-60-positive R-N87 cells, cisplatin resistance was tested three times in colony-formation assays: the 13dR-N87 (*P* = 0.0004), 7dR-N87 (*P* = 0.0018), and 3dR-N87 cells formed significantly (*P* = 0.0079) more CR colonies than the parental N87 cells (Fig. 3b, c), but the 40dR-N87 cells did not. Additionally, the 13dR-N87 cells showed significantly more CR colonies than the 3dR-N87 cells (*P* = 0.0067), suggesting that resistance to cisplatin peaks after approximately 13 days of differentiation.

Paclitaxel resistance in TRA-1-60-positive 13dR-N87 cells was also tested by colony-formation assays: the 13dR-N87 cells showed significantly more paclitaxel-resistant colonies than the parental N87 cells (*P* = 0.0022, Fig. 3d, e), indicating that 13dR-cancer cells obtained from N87 cells are also resistant to both cisplatin and paclitaxel.

### Expression profiles of H460, R-H460, 13dR-H460, and CR-13dR-H460 cells

**Fig. 4:**
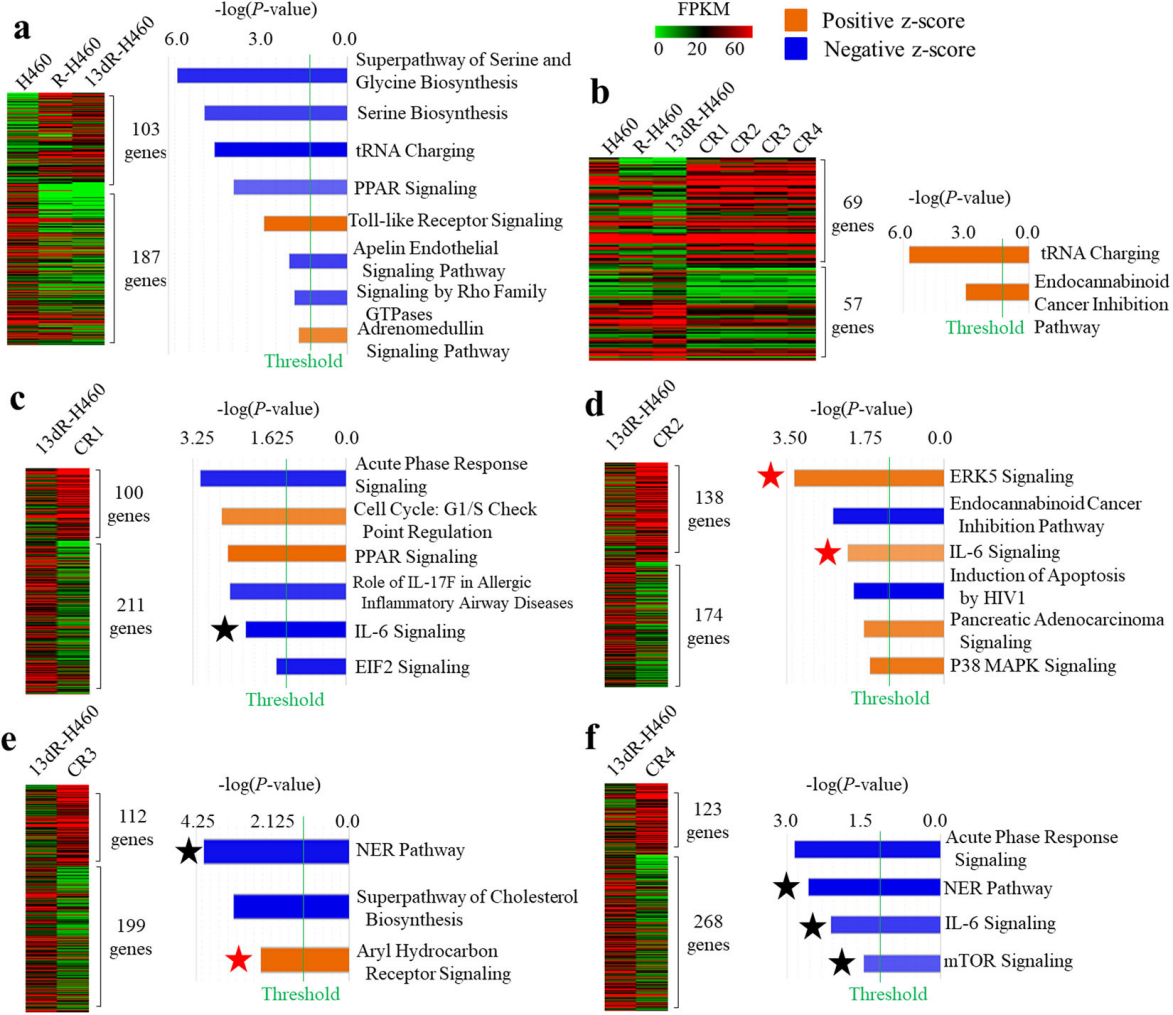
Expression profiles of parental H460, R-H460, 13dR-H460, and CR 13dR-460 clones determined by RNA sequencing. **a** Differentially expressed genes (DEGs) in H460, R-H460, and 13dR-H460 cells and their main signaling pathways. The DEGs whose expression changed more than two-fold between H460 and 13dR-H460 are shown in a heatmap produced using the Multi-Experiment Viewer. The major pathways among the DEGs were analyzed by ingenuity pathway analysis. The pathways with absolute values of z-scores and a –log (*P*-value) of at least 1.5 are shown. **b** Common DEGs and their main signaling pathways in CR clones (CR1-CR4) compared with 13dR-H460 cells. **c** DEGs specific to CR1 and the main associated signaling pathways compared with 13dR-H460 (**d**) DEGs specific to CR2. **e** DEGs specific to CR3. **f** DEGs specific to CR4. The orange- and blue-colored bars indicate predicted activation and inhibition, respectively. The red stars (Fig. 4c–f) indicate drug resistance-related signaling pathways whose levels increased in CR clones. The black stars (Fig. 4c–f) indicate drug resistance-related signaling pathways whose level was reduced in CR clones. FPKM scales for all figures are indicated.

To investigate expression profiles, RNA sequencing was performed for the H460, R-H460, 13dR-H460 cells and four CR 13dR-H460 clones (CR1-CR4). In the comparison of expression profiles between H460 and 13dR-H460 cells, we identified 290 DEGs, but most of them were also DEGs between H460 and R-H460 (Fig. 4a, left), suggesting that most DEGs in 13dR-H460 are not cisplatin resistance-related DEGs in our R-cancer model. Consistent with this, none of the signaling pathways identified in the core IPA for DEGs between H460 and 13dR-H460 cells were specifically associated with known drug resistance-related pathways (Fig. 4a, right).

In analyses of the expression profiles of the CR clones (CR-13dR-H460 clones of CR1-CR4) compared with 13dR-H460, 126 genes were common DEGs for all four CR clones. In a core IPA for the common DEGs of the four CR clones, the main signaling pathways identified were the tRNA charging and endocannabinoid cancer inhibition pathways, for which correlations with drug resistance have not been reported (Fig. 4b), suggesting that the drug resistance mechanisms of each clone may vary and may not be restricted to certain drug resistance pathways, even though they experienced the same epigenetic challenges during the experimental procedures in our R-cancer model. Therefore, each CR clone was analyzed separately.

In the analyses of the DEGs of each CR clone compared with 13dR-H460 cells (Fig. 4c–f), several candidate CR pathways were identified (marked as red or black stars in Fig. 4c–f). For example, the nuclear excision repair (NER) pathway, which has been implicated in drug resistance, specifically by the removal of cisplatin-damaged bases^19^, was consistently downregulated in the CR3 and CR4 clones. The IL-6 signaling pathway, which has also been reported to be associated with drug resistance^20^, was altered in some CR clones, although not consistently in the direction of expression-level changes: its expression level was low in CR1 and CR4 but high in CR3. Furthermore, each drug resistance-related pathway, including the ERK5 pathway^21,22^, the aryl hydrocarbon receptor pathway^23^, and the mTOR pathway^24^, was altered in one of the four CR clones, suggesting that various pathways are candidates for the induction of drug resistance via epigenetic mechanisms. The leading DEGs among the IPA pathways presented in Fig. 4 are listed in Table S1.

To analyze the role of epigenetic factors during the transition from parental H460 cells to CR clones via R-H460 and 13dR-H460 cells, 720 epigenetics-related genes (https://epifactors.autosome.ru/) were analyzed in the RNA sequencing data. Among those genes, the expression of 18 genes (Table S2) consistently was changed by more than 2-fold in both the R-H460 and 13dR-H460 cells relative to the parental H460 cells: eight of the 18 genes were related to chromatin remodeling, and another 8 genes were related to histone modifications. However, the direction of the epigenetic changes was not consistent: most factors related to chromatin remodeling were both increased and decreased, and most factors related to both increasing and decreasing histone modifications were identified simultaneously (Table S2), which might reflect the complex active process of chromatin remodeling.

In further analyses of the epigenetics-related genes between the 13dR-H460 and CR clones, the expression of 23 genes was found to be altered by more than 2-fold (Table S3). In contrast to the 18 genes whose expression changed in R-H460 and 13dR-H460 cells relative to parental H460 cells, the direction of epigenetic change for the 23 genes was relatively consistent: the expression levels of most chromatin remodeling-related genes (8/11) decreased and those of histone erasure-related genes (4/4) increased, whereas those of genes related to the introduction of histone marks (2/3) decreased (Table S3), suggesting that the potential for epigenetic change decreases during the conversion from dR-H460 cells to CR clones.

### Single-cell RNA sequencing analysis of H460, 13dR-H460, and 40dR-H460 cells

**Fig. 5:**
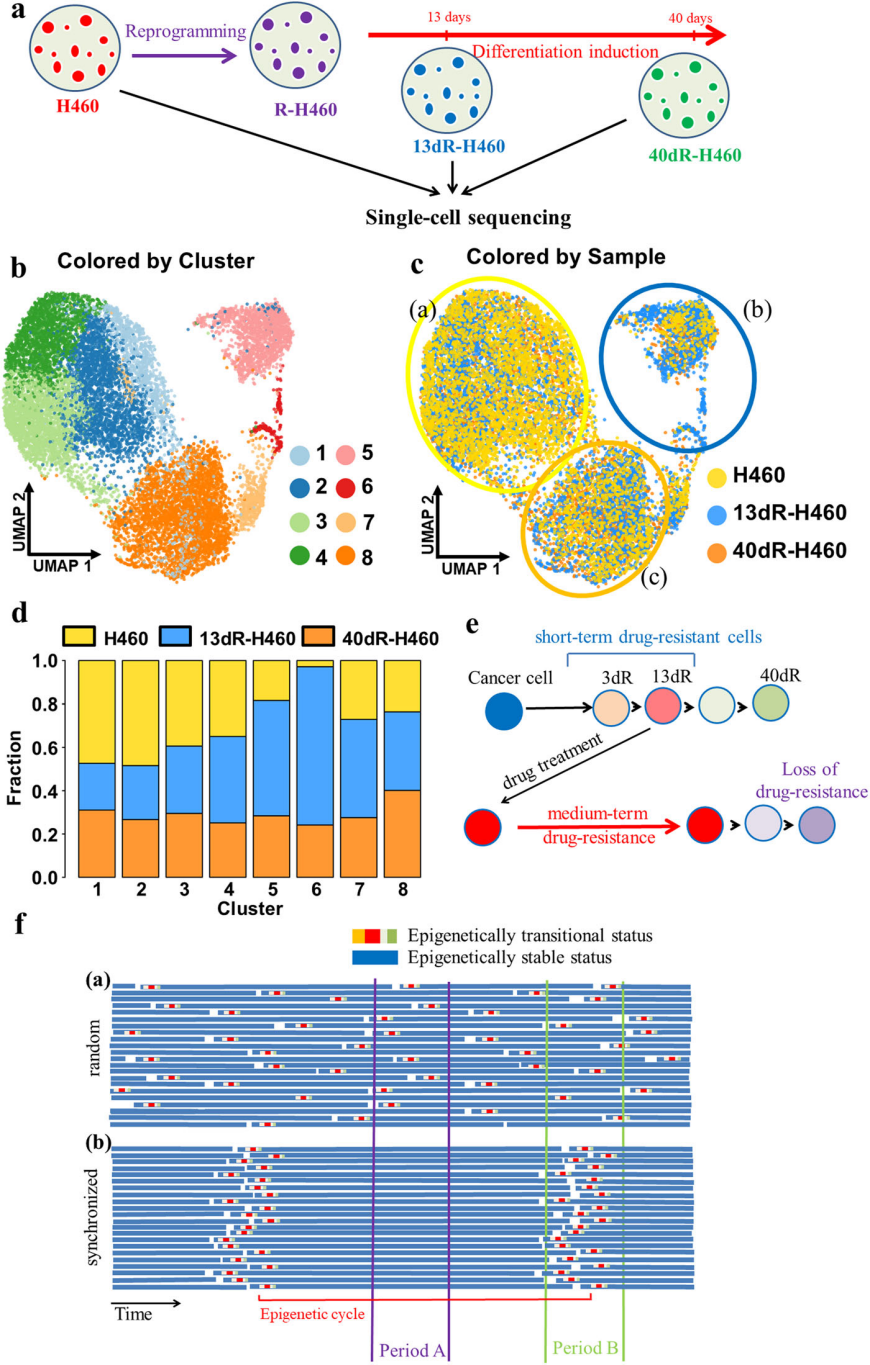
Single-cell transcriptome analysis for parental H460, 13dR-H460, and 40dR-H460 cells. **a** Experimental process for the preparation of 13dR-H460 and 40dR-H460 cells employed for single-cell transcriptome analysis. **b** UMAP plots for cluster designation based on an integrated single-cell transcriptome. Eight clusters (1 to 8) were derived from the integrated single-cell transcriptome with SEURAT. **c** UMAP plots for each sample from the integrated single-cell transcriptome. Data from H460, 13dR-H460, and 40dR-H460 are indicated by yellow, sky blue, and orange dots, respectively. H460-, 13dR-H460-, and 40dR-H460-predominant clusters are indicated by yellow, sky blue, and orange circles, respectively. Clonal heterogeneity did not change in 13dR-H460 compared to parental H460 cells; only the relative fractions changed. **d** Barplots representing the relative fractions of the three cell types in each cluster. **e** Model for epigenetically driven drug resistance based on the R-cancer system. Drug treatment of cells with short-term resistance or epigenetically transitional cells induces medium-term drug resistance. **f** Epigenetics-driven drug resistance depending on synchronized or unsynchronized epigenetic cycles. **a** With random epigenetic cycles, each cancer cell may undergo epigenetic transitions randomly, and drug treatment for period A or period B would yield a similar level of drug resistance. **b** With epigenetic cycles synchronized by the withdrawal of stem cell media, as in our R-cancer model, cancer cells may exhibit an epigenetically transitional status at the same time. Therefore, drug treatment for period A or period B would have different results depending on the amount of short-term drug-resistant cells. The Y-axis represents individual cells; the X-axis represents time progression with epigenetic cycles.

To analyze the importance of the heterogeneity of 13dR cancer cells in drug resistance, single-cell RNA sequencing was performed for H460, 13dR-H460, and 40dR-H460 cells (the scheme is shown in Fig. 5a) under the assumption that 13dR-H460 cells will contain markedly increased heterogeneous variant populations compared with parental H460 cells, which can lead to higher drug resistance.

We integrated the single-cell sequencing data from the parental H460 cells and two epigenetic H460 variants of 13dR-H460 and 40dR-H460 after quality filtration and obtained an integrated single-cell transcriptome for 14,638 cells (Fig. 5b–d). A clustering analysis revealed eight integrated cell clusters (Fig. 5b). The clusters from the integrated single-cell transcriptome were also represented in the parental H460 cells and two epigenetic H460 variants (Fig. 5c). The relative ratios of the three cell types in each cluster are shown in Fig. 5d: parental H460 cells were dominant in clusters 1–4, 13dR-H460 cells were dominant in clusters 5–7, and 40dR-H460 cells were dominant in cluster 8. These results suggested that the fractions of cells in clusters 1–4 had been converted to cells in clusters 5–7 and then eventually to cells in cluster 8 during the epigenetic transitions after stem cell medium withdrawal from R-cancer cells. Cluster 6 contained the largest fraction of 13dR-H460 cells and the smallest fraction of parental H460 cells. However, clonal heterogeneity did not increase in 13dR-H460, and only the relative fractions changed in each cluster. Therefore, our results suggest that heterogeneity might not be a significant factor in the drug resistance observed in our R-cancer model.

## Discussion

The present study results from the analysis of dR-cancer cells under anticancer drug treatments suggest an important epigenetic contribution to drug resistance. After the establishment of R-H460 or R-N87 cells by the transfection of the mRNAs of reprogramming factors (*OCT4*, *SOX2*, *KLF4*, and *MYC*), the stem cell medium was withdrawn for various periods to induce epigenetic changes: the resulting 13dR-H460 and 13dR-N87 cells, for which the stem cell medium was withdrawn for 13 days, showed peak resistance to cisplatin and paclitaxel, and this drug resistance disappeared before 40 days, indicating that dR-cancer cells show only short-term (less than a month) drug resistance, which peaks at approximately 2 weeks. Drug treatments changed the 13dR-H460 cells with short-term resistance into cells with medium-term (2–3 months) resistance, but drug resistance eventually disappeared, suggesting that the drug treatment of cancer cells with short-term resistance may cause increases in drug-resistant cells over longer periods. In single-cell analyses, the heterogeneity of 13dR-H460 cells did not increase compared with that of the parental H460 cells, but the relative ratios of each cluster did, which suggests that heterogeneity resulting from epigenetic changes is not the major epigenetic mechanism responsible for drug resistance in our R-cancer cell model.

The present study’s implication of an epigenetic mechanism in the drug resistance of 13dR cancers is consistent with previous reports^6,7,25^. First, the medium-term drug resistance caused by epigenetic mechanisms was transient: it disappeared after approximately 70–90 days of drug-free subculture in our R-cancer model. This transient medium-term drug-resistant phenotype resulting from epigenetic regulation has also been shown in the above-noted previous reports (e.g., the transient phenotype of gefitinib resistance in drug-tolerant persisters in Sharma et al. ^6^). Second, in the present study, the drug resistance of dR-cancer cells obtained from H460 or N87 cells, which peaked at approximately 13 days after differentiation, disappeared under longer differentiation, suggesting the presence of cells with short-term resistance under epigenetic changes. Additionally, our cells with short-term resistance were consistent with a previous report, which noted the disappearance of vemurafenib resistance in pre-resistant marker-sorted cancer cells after a week of culture under vemurafenib-free conditions. The marker-sorted cells showed vemurafenib resistance at 1 day after sorting^7^. Similar mechanisms have already been proposed for both cells with short-term resistance and cells with medium-term resistance^7^: Luria and Delbrück’s fluctuation analysis^26^ of patient-derived melanoma cells indicated the presence of transiently pre-resistant cells (corresponding to our cells with short-term resistance) as well as stably resistant cells (corresponding to our cells showing the development of medium-term resistance). However, we clarified that cells with short-term resistance may not be genetically selected stable cells and are probably epigenetically unstable transitional cells and showed that cells with short-term resistance may be changed into cells with medium-term resistance by drug treatment. Third, the 13dR-H460 and 13dR-N87 cells showed short-term resistance to both cisplatin and paclitaxel, suggesting that the short-term resistance phenotype caused by epigenetic mechanisms is not restricted to a specific agent but may in fact be associated with a broader range of anticancer drugs. The possibly broader drug resistance effect of the short-term resistance phenotype is similar to that observed in tolerant persisters showing epigenetically driven resistance to both gefitinib and cisplatin, according to an earlier report^6^.

Other previous reports have suggested that the epigenetically driven drug resistance mechanisms are related to the generation of heterogeneity resulting from epigenetic changes^8,27,28^; indeed, epigenetically driven markers for drug resistance have been identified, including an IGF-1 receptor inhibitor and chromatin-modifying agents^6^, or markers obtained from bulk RNA sequencing and multiplex single-cell RNA FISH^7^. Our RNA sequencing analyses of CR 13dR-H460 clones also identified several candidate marker pathways, such as the NER and IL-6 signaling pathways. However, in our single-cell analysis, the heterogeneity of 13dR-H460 cells was not increased compared with parental H460 or 40dR-H460 cells, which exhibited a basal level of cisplatin resistance, suggesting that heterogeneity may not be the primary reason for epigenetically driven drug resistance in our R-cancer cell model. Instead, the major factor underlying drug resistance may be the presence of cells with short-term resistance under epigenetic transitions. Therefore, based on our R-cancer results, previously reported epigenetic markers from drug-tolerant persisters^6^ or marker-sorted pre-resistant cells^7^ might not be related to heterogeneity but, rather, to the enrichment of cells with short-term resistance under epigenetic changes. However, our assay system with dR-cancer cells cannot be used to analyze the effect of heterogeneity on drug resistance. Therefore, epigenetically driven heterogeneity could also be an important mechanism of epigenetically driven drug resistance.

According to cancer stem cell (CSC) theory, tumor growth is fueled by a small number of plastic tumor stem cells hidden within bulky cancers after the differentiation or epigenetic modulation^29^. CSCs are also known to increase metastasis in mouse and human models^30,31^ and to increase drug resistance via various mechanisms, including the upregulation of drug-efflux pumps, a higher DNA-repair capacity, enhanced protection against ROS, or the ability to adopt a quiescent state^29,32^. Therefore, R-cancer cells are expected to show higher tumorigenesis and metastatic potential as well as drug resistance because the transfer of reprogramming factors to cancer cells can mimic plasticity and proneness to epigenetic modulation in CSCs. Consistent with this hypothesis, an earlier study showed increased metastasis of the established R-cancer cells^33^. Conversely, another study showed that in vitro sphere formation and tumor formation in mice were reduced by R-cancer cells^34^. These previously reported conflicting results regarding R-cancer cells may be related to various factors, including the instability of reprogrammed cancer cells, the remnant effects of transfected reprogramming factors, or various genetic effects of pre-existing molecular changes in cancer cells. The direct effect of the reprogramming of cancer cells needs to be investigated in further studies. We are also concerned about the direct effects of remnant R-cancer cells among prepared dR-cancer cells; however, our analysis of AP expression in various dR-cancer cells indicated that remnant R-cancer cells are not the major factor underlying the increased drug resistance of dR-cancer cells: 3dR-H460 cells, containing a small number of AP-positive cells, showed significantly lower drug resistance than the 13dR-H460 cells, containing an undetectable number of AP-positive cells.

Based on our results for dR-cancer cells as well as previously reported epigenetically driven drug resistance models^6,7,25^, we propose a revised model for epigenetically driven drug resistance, as shown in Fig. 5e, f. If one epigenetic cycle is defined as the duration from the start to the end point of an epigenetic transition in CSC-like cells in cancer tissues (Fig. 5e), the CSC-like cells will produce epigenetically transitional cells from each epigenetic cycle. Cancer cells may undergo a change from an epigenetically transitional status to a short-term resistance status, and the drug treatment of these cells with short-term resistance can lead to a medium-term drug-resistant status (Fig. 5e). If there are further genetic mutations, the medium-term drug-resistant cells will eventually become long-term or permanent drug-resistant cells. In most epigenetically unsynchronized CSC-like cancer cells, epigenetic cycles will be random, and the epigenetically driven drug resistance rate will be constant for a given time interval (Fig. 5f(a)). If the epigenetic cycles of CSC-like cancer cells can be synchronized, however, drug resistance will be dependent on the fraction of cells with short-term resistance, as shown in Fig. 5f(b): drug resistance is low in period A, during which the fraction of cells with short-term resistance is low, whereas drug resistance is high in period B, during which this fraction is high. Therefore, specific drugs suppressing short-term resistance may reduce the appearance of drug resistance. Alternatively, drugs for inducing epigenetic synchronization in cancer cells may also reduce drug resistance by the administration of combined anticancer drugs during periods such as period A in Fig. 5f(b). Therefore, our R-cancer cell model system may add another level of cancer treatment strategies to previously reported strategies employing epigenetic drugs^25^.

In conclusion, our analyses of dR-cancer cells suggest an important role of epigenetics in the drug resistance of cancer cells. Our results in this R-cancer model also suggest that cancer cells with short-term resistance, rather than heterogeneous cells, may confer epigenetically driven drug resistance. In addition, the drug treatment of cells with short-term resistance can cause more cancer cells to become resistant to drugs, which could provide insights regarding new cancer-fighting strategies involving the control of cancer cells undergoing epigenetic transition.

## Supplementary information

Supplementary Information

## References

[1] Hinds M (1991). Identification of a point mutation in the topoisomerase II gene from a human leukemia cell line containing an amsacrine-resistant form of topoisomerase II. Cancer Res..

[2] Kobayashi S (2005). EGFR mutation and resistance of non-small-cell lung cancer to gefitinib. N. Engl. J. Med..

[3] Zhang N, Yin Y, Xu SJ, Chen WS (2008). 5-Fluorouracil: mechanisms of resistance and reversal strategies. Molecules.

[4] Berns K (2007). A functional genetic approach identifies the PI3K pathway as a major determinant of trastuzumab resistance in breast cancer. Cancer Cell.

[5] Gorre ME (2001). Clinical resistance to STI-571 cancer therapy caused by BCR-ABL gene mutation or amplification. Science.

[6] Sharma SV (2010). A chromatin-mediated reversible drug-tolerant state in cancer cell subpopulations. Cell.

[7] Shaffer SM (2017). Rare cell variability and drug-induced reprogramming as a mode of cancer drug resistance. Nature.

[8] Easwaran H, Tsai HC, Baylin SB (2014). Cancer epigenetics: tumor heterogeneity, plasticity of stem-like states, and drug resistance. Mol. cell.

[9] Semi K, Yamada Y (2015). Induced pluripotent stem cell technology for dissecting the cancer epigenome. Cancer Sci..

[10] Warren L (2010). Highly efficient reprogramming to pluripotency and directed differentiation of human cells with synthetic modified mRNA. Cell Stem cell.

[11] Chang X (2010). Identification of hypermethylated genes associated with cisplatin resistance in human cancers. Cancer Res..

[12] Bomane A, Goncalves A, Ballester PJ (2019). Paclitaxel response can be predicted with interpretable multi-variate classifiers exploiting DNA-methylation and miRNA Data. Front. Genet..

[13] Jang H (2012). O-GlcNAc regulates pluripotency and reprogramming by directly acting on core components of the pluripotency network. Cell Stem Cell.

[14] Kim, Y. H. et al. FAK-copy-gain is a predictive marker for sensitivity to FAK inhibition in breast cancer. *Cancers* **11**, 1288 (2019). 10.3390/cancers11091288 PMC6769494 31480645

[15] Kim HY (2018). Farnesyl diphosphate synthase is important for the maintenance of glioblastoma stemness. Exp. Mol. Med..

[16] Zheng GX (2017). Massively parallel digital transcriptional profiling of single cells. Nat. Commun..

[17] Butler A, Hoffman P, Smibert P, Papalexi E, Satija R (2018). Integrating single-cell transcriptomic data across different conditions, technologies, and species. Nat. Biotechnol..

[18] Becht, E. et al. Dimensionality reduction for visualizing single-cell data using UMAP. *Nature Biotechnol.* **37**, 38–44 (2018). 10.1038/nbt.4314 30531897

[19] Bonanno L, Favaretto A, Rosell R (2014). Platinum drugs and DNA repair mechanisms in lung cancer. Anticancer Res..

[20] Duan S (2015). IL-6 signaling contributes to cisplatin resistance in non-small cell lung cancer via the up-regulation of anti-apoptotic and DNA repair associated molecules. Oncotarget.

[21] Stecca, B. & Rovida, E. Impact of ERK5 on the Hallmarks of Cancer. *Int. J. Mol. Sci.* **20**, 1426 (2019). 10.3390/ijms20061426 PMC6471124 30901834

[22] Hoang VT (2017). MEK5-ERK5 signaling in cancer: implications for targeted therapy. Cancer Lett..

[23] Ye M (2018). Activation of the aryl hydrocarbon receptor leads to resistance to EGFR TKIs in non-small cell lung cancer by activating Src-mediated bypass signaling. Clin. Cancer Res..

[24] Liang SQ (2019). mTOR mediates a mechanism of resistance to chemotherapy and defines a rational combination strategy to treat KRAS-mutant lung cancer. Oncogene.

[25] Brown R, Curry E, Magnani L, Wilhelm-Benartzi CS, Borley J (2014). Poised epigenetic states and acquired drug resistance in cancer. Nat. Rev. Cancer.

[26] Luria SE, Delbruck M (1943). Mutations of bacteria from virus sensitivity to virus resistance. Genetics.

[27] Hoey T (2010). Drug resistance, epigenetics, and tumor cell heterogeneity. Sci. Transl. Med..

[28] Wilting RH, Dannenberg JH (2012). Epigenetic mechanisms in tumorigenesis, tumor cell heterogeneity and drug resistance. Drug Resist. Updates.

[29] Batlle E, Clevers H (2017). Cancer stem cells revisited. Nat. Med..

[30] Celia-Terrassa T, Kang Y (2016). Distinctive properties of metastasis-initiating cells. Genes Dev..

[31] Lawson DA (2015). Single-cell analysis reveals a stem-cell program in human metastatic breast cancer cells. Nature.

[32] Phi LTH (2018). Cancer stem cells (CSCs) in drug resistance and their therapeutic implications in cancer treatment. Stem Cells Int..

[33] Singovski G (2016). In vivo epigenetic reprogramming of primary human colon cancer cells enhances metastases. J. Mol. Cell Biol..

[34] Khoshchehreh R (2019). Epigenetic reprogramming of primary pancreatic cancer cells counteracts their in vivo tumourigenicity. Oncogene.

