# Supplementary material for: Cancer cells undergoing epigenetic transition show short-term resistance and are transformed into cells with medium-term resistance by drug treatment
Source: Exp Mol Med. 2020 Jul 13;52(7):1102–15. doi: 10.1038/s12276-020-0464-3 (PMC8080688; doi:10.1038/s12276-020-0464-3)
Supplement: Supplementary file 1 — Supplementary Information [file 12276_2020_464_MOESM1_ESM.pdf]

Supplementary Information

Epigenetically transitional cancer cells show short-term resistance and change into medium-term-resistant cells by drug treatment

Shiv Poojan[1], Seung-Hyun Bae[1,2], Jae-Woong Min[3-5], Eun Young Lee [1], Yura Song[1], Hee Yeon Kim[1], Hye Won Sim[1], Eun-Kyung Kang[1], Young-Ho Kim[1], Hae-Ock Lee[3-5], Yourae Hong[3-5], Woong-Yang Park[3-5], Hyonchol Jang[1,2,*], Kyeong-Man Hong[1,*]

List of Supplementary Information

Figure S1. Comparison of our protocol and the original mRNA transfection protocol for preparation of reprogramming cells.

Table S1. Leading differentially expressed genes in each biological pathway described in Figure 4.

Table S2. Epigenetics-related DEGs among H460, R-H460, and 13dR.

Table S3. Epigenetics-related DEGs among 13dR and CR1-4.

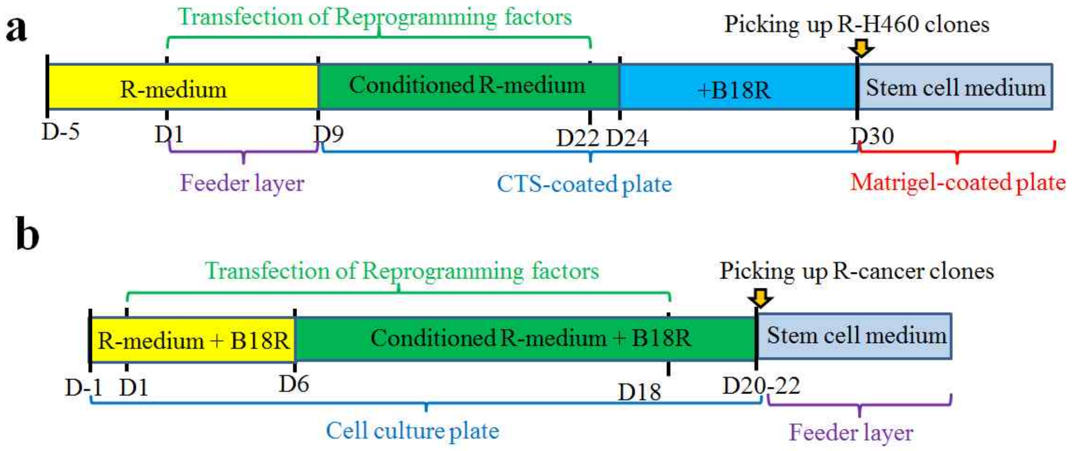

Figure S1. Comparison of our protocol with the original mRNA transfection protocol for preparation of reprogramming cells. a. Protocol for reprogramming of cancer cells in the present study. b. Original protocol of Stemgent mRNA reprogramming kit. We did not obtain any reprogrammed cancer cells with the original protocol from the Stemgent mRNA reprogramming kit, and had to modify our approach. The first modification was to use feeder cells at the first stage of transfection. When the feeder layer was maintained until D22, the identification of reprogrammed cells was difficult due to the co-existing feeder cells, and we had to change the plate by employing a CTS-coated plate to support the growth of stem cells[1]. B18R is employed for the reduction of cell damage during transfection[2], but it has been shown to be deleterious to cancer cell reprogramming. Therefore, we did not use B18R during the transfection of reprogramming factors, but it was included in the media once the transfection was finished.

REFERENCES

1. Kaur, J., Tilkins, M.L., Eckert, R. & Boucher, S. Methods for culturing human embryonic stem cells in a xeno-free system. Methods in molecular biology 997, 115-126 (2013).

2. Warren, L., et al. Highly efficient reprogramming to pluripotency and directed differentiation of human cells with synthetic modified mRNA. Cell stem cell 7, 618-630 (2010).

Table S1. Leading Differentially expressed genes in each biological pathway described in Figure 4.

Figure 4a				
Ingenuity Canonical Pathways	-log(p-value)	Ratio	z-score	Molecules
Superpathway of Serine and Glycine Biosynthesis I	5.92	0.263	-2.236	NAD ,PHGDH,PSAT1,PSPH,SHMT2
Serine Biosynthesis	4.98	0.286	-2	NAD ,PHGDH,PSAT1,PSPH
tRNA Charging	4.63	0.0854	-2.646	AARS,CARS,EPRS,GARS,MARS,SARS,YARS
PPAR Signaling	3.96	0.0673	-1.633	FOS,IL18,IL1B,NFKBIA,NR2F1,PDGFA,PTGS2
Toll-like Receptor Signaling	2.9	0.0649	2	FOS,IL18,IL1B,MAP2K6,NFKBIA
Apelin Endothelial Signaling Pathway	2.03	0.0407	-2	APLN,FOS,KLF2,MEF2C,PRKAA2
Signaling by Rho Family GTPases	1.84	0.0286	-1.89	ARHGEF2,CDC42EP1,CDH10,CDH11,FOS,PAK3,RHOH
Adrenomedullin signaling pathway	1.69	0.0293	1.633	FOS,IL18,IL1B,MAP2K6,NPR1,PRKAR1B

Figure 4b				
Ingenuity Canonical Pathways	-log(p-value)	Ratio	z-score	Molecules
tRNA Charging	5.76	0.128	2.236	AARS,CARS,GARS,MARS,SARS
Endocannabinoid Cancer Inhibition Pathway	3.04	0.035	2.236	CASP4,CCND3,CCNE2,PRKAR1B,TRIB3

Figure 4c				
Ingenuity Canonical Pathways	-log(p-value)	Ratio	z-score	Molecules
Acute Phase Response Signaling	2.38	0.0497	-1.414	C1S,FGG,HRAS,IL1B,IL6,JUN,NFKBIA,NFKBIB,TNFRSF11B
Cell Cycle: G1/S Checkpoint Regulation	4.73	0.119	2.646	BMI1,CCND3,CCNE2,CDC25A,CDK4,CDKN1B,E2F4,PA2G4
PPAR Signaling	2.68	0.0673	1.89	CITED2,HRAS,IL1B,JUN,NFKBIA,NFKBIB,TNFRSF11B
Role of IL-17F in Allergic Inflammatory Airway Diseases	3	0.111	-2.236	CXCL1,CXCL5,IL1B,IL6,RPS6KA4
IL-6 Signaling	2.23	0.056	-1.89	HRAS,IL1B,IL6,JUN,NFKBIA,NFKBIB,TNFRSF11B
EIF2 Signaling	1.77	0.0396	-2.449	DDIT3,EIF2B1,EIF4A2,EIF4G1,EIF5,HRAS,HSPA5,TRIB3,WARS

Figure 4d				
Ingenuity Canonical Pathways	-log(p-value)	Ratio	z-score	Molecules
ERK5 Signaling	2.69	0.0833	2.236	BAD,ELK4,FOSL1,LIF,MYC,SH2D2A
Endocannabinoid Cancer Inhibition Pathway	4.89	0.0839	0	BAD,CASP2,CASP4,CASP9,CCND3,CCNE2,DDIT3,GNAI2,MYC,PRKAR1B,TRIB3,VEGFA
IL-6 Signaling	2.09	0.056	1.89	CEBPB,CXCL8,IL1A,IL1RAP,JUN,TNFRSF11B,VEGFA
Induction of Apoptosis by HIV1	1.6	0.0656	-2	BCL2L1,CASP9,SLC25A10,TNFRSF11B
Pancreatic Adenocarcinoma Signaling	1.3	0.0459	2	BAD,BCL2L1,CASP9,E2F1,VEGFA
p38 MAPK Signaling	1.19	0.0424	2.236	DDIT3,IL1A,IL1RAP,MKNK2,MYC

Figure 4e				
Ingenuity Canonical Pathways	-log(p-value)	Ratio	z-score	Molecules
NER Pathway	4.71	0.0971	-2.121	CHAF1A,CHAF1B,DNA2,LIG1,PCNA,POLA2,POLE2,POLR2L,RPA2,TCEA1
Superpathway of Cholesterol Biosynthesis	2.8	0.143	-2	DHCR24,EBP,IDI1,LSS
Aryl Hydrocarbon Receptor Signaling	4.88	0.0839	0.632	ALDH1A1,ALDH1L2,CCND3,CCNE2,DHFR,E2F1,HSP90AA1,HSP90AB1,IL1A,JUN,NCOA3,TGM2

Figure 4f				
Ingenuity Canonical Pathways	-log(p-value)	Ratio	z-score	Molecules
Acute Phase Response Signaling	2.4	0.0552	-1.667	FGG,HMOX1,HRAS,IL18,IL1A,IL6,JUN,NOLC1,SOCS3,TNFRSF11B
NER Pathway	3.48	0.0841	-2.646	CHAF1A,CHAF1B,PCNA,POLA1,POLA2,POLE2,POLE3,RPA2,SLC19A1
IL-6 Signaling	2.4	0.064	-1.414	HRAS,IL18,IL1A,IL6,JUN,MAP4K4,SOCS3,TNFRSF11B
mTOR Signaling	1.89	0.0463	-1.134	DDIT4,EIF3B,EIF4EBP1,EIF4G1,HMOX1,HRAS,PLD3,RND3,RPS6KA4,ULK1

Table S2. Epigenetics-related DEGs among H460, R-H460, and 13dR.

Function	Gene	H460	R-H460	13dR
Chromatin remodeling	HMGN3	5.97004	4.4444	10.5138
Chromatin remodeling	CDC6	12.1756	16.7383	25.9572
Chromatin remodeling	CHD4	3.29526	12.0483	10.2875
Chromatin remodeling	DEK	24.208	16.886	10.4529
Chromatin remodeling	GADD45A	20.9768	6.96703	8.94347
Chromatin remodeling	SAFB	7.8327	0.336309	7.67378
Chromatin remodeling cofactor, Histonemodificationwritecofactor	PHF19	13.9138	6.76201	13.9072
Chromatin remodeling, TF	NPAS2	2.87379	5.87132	4.44163
Histone chaperone	POLE3	14.6169	6.33166	18.679
Histone modification cofactor	PPARGC1A	0.348648	7.33559	0.592676
Histone modification erase	KDM4A	5.49581	3.43444	2.53637
Histone modification erase	KDM5C	7.30482	7.10172	0.867726
Histone modification erase	PPP4C	3.65472	3.82133	0.677145
Histone modification erase cofactor	MORF4L2	5.74684	24.2974	8.68326
Histone modification read	WDR5	3.73232	0.362079	4.08824
Histone modification write	FBL	4.30055	1.742	2.79287
Histone modification write	PRMT1	18.5976	9.24885	16.7376
Histone modification write cofactor	DDB2	2.93059	3.31134	5.99405
Histone modification write cofactor, Histone modification erase cofactor	DNAJC1	5.85147	12.2002	6.3161
Histone modification write cofactor	HSPA1A	3.32746	21.9976	12.447
Polycomb group (PcG) protein	PHC2	16.5536	7.46646	10.2231

Among 720 epigenetics-related genes (https://epifactors.autosome.ru/), the differentially expressed genes (DEGs) among H460, R-H460, and 13dR were represented. Genes whose expression changed more than two-fold in R-H460 or 13dR compared with H460 are shown. Gene less than 5 FPKM in all samples were excluded. Numbers represent FPKM. Red : Upregulated genes; Blue : Downregulated genes.

Table S3. Epigenetics-related DEGs among 13dR and CR1-4.

Function	Gene	13dR	CR1	CR2	CR3	CR4
Chromatin remodeling	HMGN3	10.5138	4.07347	1.41204	1.37221	0.049122
Chromatin remodeling	CDC6	25.9572	8.11086	9.77474	8.85442	10.0641
Chromatin remodeling	CHD3	11.1171	5.41278	1.76075	6.83655	6.221
Chromatin remodeling	DEK	10.4529	19.9193	7.18217	7.37041	33.7957
Chromatin remodeling	GADD45A	8.94347	12.5174	37.2532	22.9313	13.6665
Chromatin remodeling	LRWD1	9.20302	3.96127	6.441	6.58948	5.91011
Chromatin remodeling	PSIP1	16.0363	7.04796	3.89316	16.143	18.0946
Chromatin remodeling	SAFB	7.67378	4.1927	3.57316	8.98116	2.42103
Chromatin remodeling	TONSL	5.22612	1.90052	1.91242	1.42707	1.97701
Chromatin remodeling cofactor	SMARCD2	14.7547	6.28921	14.9535	15.7385	7.36401
Chromatin remodeling, TF	NPAS2	4.44163	9.98984	4.42391	5.05063	10.258
DNA modification cofactor	APEX1	93.3488	47.3476	39.584	77.6917	24.3717
RNA modification	SRSF1	48.1141	20.3741	28.7084	34.4375	21.176
Histone chaperone	POLE3	18.679	6.46028	13.6852	12.952	5.97326
Histone chaperone, Histone modification read	ANP32E	8.19034	7.16667	5.36372	4.06487	5.6489
Histone modification	SET	12.825	3.79898	6.60049	5.33315	2.91753
Histone modification erase	KDM4A	2.53637	4.96117	5.26191	4.72105	4.46607
Histone modification erase	KDM5C	0.867726	7.94183	0.895037	13.4203	0.774498
Histone modification erase	PPP4C	0.677145	3.10675	2.84504	1.53038	14.0794
Histone modification erase cofactor	UCHL5	0.941261	0.189601	11.9902	7.15887	1.41578
Histone modification write	FBL	2.79287	2.00478	6.23751	2.85197	2.15847
Histone modification write	PKN1	9.5457	9.25308	9.12684	3.43029	8.68615
Histone modification write cofactor	HSPA1A	12.447	4.70718	6.65862	4.23716	9.41208

Among 720 epigenetics-related genes (https://epifactors.autosome.ru/), the differentially expressed genes (DEGs) among 13dR and CR1-4 were represented. Genes whose expression changed more than two-fold in CR1-4 compared with 13dR are shown. Gene less than 5 FPKM in all samples were excluded. Numbers represent FPKM. Red: Upregulated genes; Blue: Downregulated genes.
